# Neutrophil-to-lymphocyte ratio predicts critical illness patients with 2019 coronavirus disease in the early stage

**DOI:** 10.1186/s12967-020-02374-0

**Published:** 2020-05-20

**Authors:** Jingyuan Liu, Yao Liu, Pan Xiang, Lin Pu, Haofeng Xiong, Chuansheng Li, Ming Zhang, Jianbo Tan, Yanli Xu, Rui Song, Meihua Song, Lin Wang, Wei Zhang, Bing Han, Li Yang, Xiaojing Wang, Guiqin Zhou, Ting Zhang, Ben Li, Yanbin Wang, Zhihai Chen, Xianbo Wang

**Affiliations:** 1 Critical Care Medicine Department, Beijing Ditan Hospital, Capital Medical University, No. 8 Jing Shun East Street, Beijing, 100015 China; 2 Center of Integrative Medicine, Beijing Ditan Hospital, Capital Medical University, No. 8 Jing Shun East Street, Beijing, 100015 China; 3 Center of Infectious Diseases, Beijing Ditan Hospital, Capital Medical University, No. 8 Jing Shun East Street, Beijing, 100015 China; 4 Liver Diseases Center, Beijing Ditan Hospital, Capital Medical University, No. 8 Jing Shun East Street, Beijing, 100015 China

**Keywords:** COVID-19, 2019-nCoV, NLR, Model, Prognosis, SARS-CoV

## Abstract

**Background:**

Patients with critical illness due to infection with the 2019 coronavirus disease (COVID-19) show rapid disease progression to acute respiratory failure. The study aimed to screen the most useful predictive factor for critical illness caused by COVID-19.

**Methods:**

The study prospectively involved 61 patients with COVID-19 infection as a derivation cohort, and 54 patients as a validation cohort. The predictive factor for critical illness was selected using LASSO regression analysis. A nomogram based on non-specific laboratory indicators was built to predict the probability of critical illness.

**Results:**

The neutrophil-to-lymphocyte ratio (NLR) was identified as an independent risk factor for critical illness in patients with COVID-19 infection. The NLR had an area under receiver operating characteristic of 0.849 (95% confidence interval [CI], 0.707 to 0.991) in the derivation cohort and 0.867 (95% CI 0.747 to 0.944) in the validation cohort, the calibration curves fitted well, and the decision and clinical impact curves showed that the NLR had high standardized net benefit. In addition, the incidence of critical illness was 9.1% (1/11) for patients aged ≥ 50 and having an NLR < 3.13, and 50% (7/14) patients with age ≥ 50 and NLR ≥ 3.13 were predicted to develop critical illness. Based on the risk stratification of NLR according to age, this study has developed a COVID-19 pneumonia management process.

**Conclusions:**

We found that NLR is a predictive factor for early-stage prediction of patients infected with COVID-19 who are likely to develop critical illness. Patients aged ≥ 50 and having an NLR ≥ 3.13 are predicted to develop critical illness, and they should thus have rapid access to an intensive care unit if necessary.

## Background

Coronavirus is a large virus family, members of which are known to cause common cold and serious illnesses, such as the Middle East respiratory syndrome and severe acute respiratory syndrome [1–4]. It was found that the 2019 coronavirus disease (COVID-19) was the cause of unexplained viral pneumonia in Wuhan, China in December 2019, and this virus was recognized by the World Health Organization on January 12, 2020. In the following month, COVID-19 was reported to spread throughout the Hubei Province and China and even to other countries [5], causing 34,662 confirmed cases of infection by February 8, 2020.

Most patients infected with the novel coronavirus had mild and moderate illness, and severe illness often experienced dyspnea after 1 week. In cases of critical illness, patients progressed rapidly to acute respiratory failure, acute respiratory distress syndrome, metabolic acidosis, coagulopathy, and septic shock. Early identification of risk factors for critical illness facilitated appropriate provision of supportive care and rapid access to the intensive care unit (ICU) when required. For patients with mild and moderate illness, general isolation treatment is required and ICU-care is not needed unless the condition worsens. Thus, early prognosis prediction would help reduce mortality and alleviate the shortage of medical resources.

Of note, a high incidence of lymphopenia in COVID-19 patients has been reported by Cao and his colleagues [6]. In addition, the baseline neutrophil-to-lymphocyte ratio (NLR) has been confirmed as a potential short-term prognostic indicator for patients with acute-on-chronic hepatitis B liver failure [7]. Thus, we wondered that whether NLR might be a potential predictor for critical illness of COVID-19. To test this hypothesis, we included 26 variables including NLR along with epidemiological history, comorbidity, and other laboratory tests for LASSO regression analysis.

## Methods

### Patient selection

This study was a prospective single-center study, which included 61 patients with COVID-19 infection treated at Beijing Ditan Hospital from January 13 to 31, 2020 as a derivation cohort, and 54 patients included from February 1 to 24, 2020 as a validation cohort. The inclusion criteria are as follows: (1) confirmed cases of COVID-19, which was diagnosed based on the new coronavirus pneumonia diagnosis and treatment plan (trial version 5) developed by the National Health Committee of the People’s Republic of China (http://www.nhc.gov.cn/). The diagnostic criteria are as follows: epidemiological history: within 14 days before the onset of COVID-19, there were travel history or residential history in Wuhan or surrounding areas, contact history of people with COVID-19, contact history of people with fever or respiratory symptoms from Wuhan or surrounding areas, contact history of people with fever or respiratory symptoms from the community which was confirmed to have COVID-19 cases; clinical manifestations: fever and/or respiratory symptoms, imaging characteristics of pneumonia, leukocyte count was normal or decreased, or the lymphocyte count was decreased. Etiological evidence: real-time polymerase chain reaction test of respiratory or blood samples was positive for the nucleic acid of COVID-19, and the viral gene sequencing of respiratory or blood samples was highly homologous with the known COVID-19. Confirmed case: any one of the epidemiological history, any two of the clinical manifestations, and any one of the etiological evidence; if there is no clear epidemiological history, any three of the clinical manifestations, and any one of the etiological evidence. (2) complete baseline characteristics at the onset time. The exclusion criterion was primary infection by influenza virus, parainfluenza virus, adenovirus, respiratory syncytial virus, rhinovirus, human metapneumovirus, SARS coronavirus, mycoplasma, chlamydia and bacteria. Clinical classification of COVID-19 pneumonia were done according to the new coronavirus pneumonia diagnosis and treatment plan (trial version 5) developed by the National Health Committee of the People’s Republic of China (http://www.nhc.gov.cn/). The clinical classifications are as follows: (1) mild, slight clinical symptoms, no pneumonia manifestations on imaging. (2) moderate, with fever, respiratory tract symptoms, and imaging shows pneumonia. (3) severe, meet any of the following: (a) respiratory distress, respiratory rate ≥ 30 beats/min; (b) in the resting state, mean oxygen saturation ≤ 93%; (c) arterial blood oxygen partial pressure/oxygen concentration ≤ 300 mmHg (1 mmHg = 0.133 kPa). (4) critical, meets any of the following: (a) respiratory failure occurs and requires mechanical ventilation; (b) Shock occurs; (c) ICU admission is required for combined organ failure.

### Data at onset

All screened COVID-19 infection patients had upper respiratory tract (throat swab) samples taken upon admission; these samples were stored in virus transport medium and then transported to Beijing Center for Disease Control and Prevention for laboratory diagnosis by real-time polymerase chain reaction test. Influenza A virus (H1N1, H3N2, H7N9), influenza B virus, bacterium, and fungus detection in sputum or respiratory secretions was performed. Epidemiological history, comorbidity, vital signs, symptoms, signs, laboratory tests, including biochemical indicators, blood routine, C-reactive protein, chest radiograph, or CT scan were collected at onset time of COVID-19.

### Follow-up

After admission, the patients were re-examined for laboratory indexes and imaging analysis, and recorded symptoms and signs, treatments and outcome events. The endpoint of this study was the development of critical illness.

### Statistical analysis

Age and days were represented as median (range), categorical variables by number (%), and laboratory data by mean (interquartile range). The cutoff values of neutrophil-to-lymphocyte ratio (NLR) and age were calculated based on the maximum Youden index (sensitivity + specificity − 1). Comparison of the differences between the two cohorts was conducted using the t-test, Chi-square test, or Mann–Whitney U test. Multivariate Cox proportional hazards regression analyses (stepwise forward method) were performed to identify the most useful predictive factor for predicting critical illness incidence. p-value < 0.05 was considered statistically significant. Analyses were performed using SPSS 22.0 statistical package (SPSS, Inc., Chicago, IL, USA).

R software version 3.6.3 (R Foundation for Statistical Computing, Vienna, Austria) was used to establish LASSO regression analysis, nomogram, Harrell’ concordance index, calibration, decision and clinical impact curves. LASSO regression is a type of machine learning regression, which was used to select independent risk factors that affect outcomes. The regression was generated using the glmnet package in R. Harrell’s concordance index is routinely used to measure how well a variable or model predicts the time to a censored event. The index was generated using the rms package in R [8]. The calibration curve reflects the relationship between the prediction rate and the actual occurrence rate. The curve was also generated using the rms package in R. The abscissa is the prediction probability. The prediction model is used to predict the probability of the event, and 0 to 1 means the probability of the event is 0 to 100%. The ordinate is the actual probability (actual incident rate) of the patient. The red line is the fit line, which represents the actual value corresponding to the predicted value [9]. The decision curve is a useful tool to evaluate the clinical application of the model, which displays estimates of the standardized net benefit by the probability threshold used to categorize observations as ‘high risk.’ The clinical impact curve is an alternative plot for the output of the decision curve. Decision and clinical impact curves were generated using the DecisionCurve package in R [10].

## Results

### Derivation and validation cohort characteristics

Of the 61 patients with COVID-19 infection included in the derivation cohort, the infections of 44 (72.1%) were diagnosed as mild or moderate and those of 17 (27.9%) were diagnosed as severe or critical on admission. In the validation cohort, the infections of 34 (63.0%) were diagnosed as mild or moderate and those of 20 (37.0%) were diagnosed as severe or critical. None of the 115 patients had a history of Huanan seafood market exposure in Wuhan. 44 of patients (72.1%) with pneumonia caused by COVID-19 infection in the derivation cohort were Wuhan citizens or visited Wuhan recently, but 44 of patients (81.5%) in the validation cohort had not left Beijing recently, but had a close exposure history with COVID-19. There was no significant difference between the two cohorts in terms of comorbidity (Table 1). Among the 61 patients in the derivation cohort, 5 (8.2%) had high fever (> 39 °C), 3 (4.9%) had dyspnea. 7 (11.5%) had mild shortness of breath. 11 (18.0%) patients had gastrointestinal symptoms. The laboratory test showed that the white blood cell count, neutrophil count, lymphocyte count, and platelet count in the validation cohort was significantly higher than that in the derivation cohort (Table 1).

**Table 1:** Demographics and characteristics of patients infected with COVID-19

	Derivation cohort (n = 61)	Validation cohort (n = 54)	p value
Characteristics			
Age, years	40 (1–86)	45 (1–92)	0.983
Gender			0.268
Male	31 (50.8)	33 (61.1)	
Female	30 (49.2)	21 (38.9)	
Current smoking	4 (6.6)	6 (11.1)	0.387
Drinking	13 (21.3)	12 (22.2)	0.906
Exposure			
Wuhan residents come to Beijing	44 (72.1)	10 (18.5)	< 0.0001
Comorbidity			
Diabetes	5 (8.2)	5 (9.3)	0.840
Hypertension	12 (19.7)	13 (24.1)	0.568
Cardiovascular disease	1 (1.6)	3 (5.6)	0.253
Chronic obstructive pulmonary disease	5 (8.2)	1 (1.9)	0.127
Disease type on admission this hospital			0.071
Mild	5 (8.2)	9 (16.7)	
Moderate	39 (63.9)	25 (46.3)	
Severe	14 (23.0)	15 (27.8)	
Critical	3 (4.9)	5 (9.3)	
Signs and symptoms			
Highest temperature, °C			
37.3–38.0	21 (34.4)	13 (24.1)	0.225
38.1–39.0	34 (55.7)	25 (46.3)	0.312
> 39.0	5 (8.2)	4 (7.4)	0.875
Dyspnea	3 (4.9)	4 (7.4)	0.577
Mild shortness of breath	7 (11.5)	12 (22.2)	0.121
Cough	39 (63.9)	38 (70.4)	0.464
Sputum production	27 (44.3)	22 (40.7)	0.703
Fatigue	35 (57.4)	26 (48.1)	0.322
Headache	21 (34.4)	6 (11.1)	0.003
Chill	12 (19.7)	9 (16.7)	0.677
Anorexia	8 (13.1)	6 (11.1)	0.743
Nausea or vomiting	5 (8.2)	5 (9.3)	0.840
Diarrhea	6 (9.8)	2 (3.7)	0.197
Sore throat	10 (16.4)	8 (14.8	0.816
Chest pain	1 (1.6)	0 (0)	0.345
Systolic pressure < 90 or diastolic pressure ≤ 60, mmHg	5 (8.2)	6 (11.1)	0.596
Respiratory rate > 30 breaths per min	2 (3.3)	4 (7.4)	0.320
Blood laboratory findings			
White blood cell count, ×10^9^/L	4.3 (3.5–5.1)	5.4 (4.1–7.0)	< 0.0001
Neutrophil count, ×10^9^/L	2.5 (2.1–3.5)	3.0 (2.1–4.6)	0.036
Lymphocyte count, ×10^9^/L	1.0 (0.8–1.4)	1.3 (1.0–1.9)	0.011
Monocyte count, ×10^9^/L	0.3 (0.2–0.4)	0.3 (0.2–0.4)	0.079
NLR	2.6 (1.6–3.5)	2.3 (1.5–3.9)	0.676
C-reactive protein, mg/L	12.0 (3.7–27.8)	21.6 (1.9–87.4)	0.184
Hemoglobin, g/L	138.0 (127.0–150.5)	139.0 (127.8–147.0)	0.773
Platelet count, ×10^9^/L	164.0 (135.0–219.5)	205.5 (149.8–263.6)	0.013
Prothrombin time, s	12.0 (11.1–13.1)	12.2 (11.8–13.1)	0.191
Potassium, mmol/L	3.8 (3.5–4.1)	3.8 (3.6–4.2)	0.408
Sodium, mmol/L	139.0 (137.0–140.0)	138.5 (136.4–139.7)	0.112
Serum Chlorine, mmol/L	102.0 (100.0–104.0)	102.3 (100.2–105.3)	0.796
Serum urea nitrogen, mmol/L	4.3 (3.5–5.6)	4.3 (3.4–5.5)	0.989
Creatinine, μmol/L	60.0 (47.0–69.5)	69.3 (51.6–80.1)	0.069
Serum glucose, mmol/L	6.1 (5.5–6.9)	5.9 (5.2–7.7)	0.407
Creatine kinase, U/L	93.0 (57.0–137.0)	89.4 (63.7–141)	0.643
Alanine aminotransferase, U/L	19.0 (14.0–33.5)	23.7 (13.9–35.4)	0.295
Albumin, g/L	44.0 (40.5–47.0)	41.1 (36.2–44.2)	0.005
Multiple lung lobe or bilateral involvement	48 (78.7)	37 (68.5)	0.743
With bacterial infection	8 (13.1)	6 (11.1)	0.231
Timeline after onset of illness, median (range)			
Days from illness onset to admission time	5 (0–23)	7 (0–21)	0.042
Days from illness onset to dyspnea	3 (2–11)	7 (0–9)	0.906
Days from illness onset to ICU admission	9 (2–14)	10 (4–14)	0.643
Treatment			
Antiviral therapy	34 (55.7)	36 (66.7)	0.231
Antibiotic therapy	26 (42.6)	21 (38.9)	0.684
Use of corticosteroid	2 (3.3)	3 (5.6)	0.550
Oxygen support	20 (32.8)	24 (44.4)	0.199
Nasal cannula	15 (24.6)	19 (35.2)	
Non-invasive ventilation	3 (4.9)	2 (3.7)	
Invasive mechanical ventilation	2 (3.3)	3 (5.6)	
Nebulization inhalation	52 (85.2)	49 (90.7)	0.368
Outcomes			
Dead	0 (0)	1 (1.9)	0.286
Transfer to ICU	8 (13.1)	6 (11.1)	0.743
Discharge	3 (4.9)	19 (35.2)	< 0.0001
Hospitalization	50 (82.0)	28 (51.9)	0.001

Data are median (range), n (%), or median (interquartile range)

*COVID-19* 2019 novel coronavirus, *NLR* neutrophil-to-lymphocyte ratio, *NA* not applicable, *ICU* intensive care unit

p values comparing mild group and severe group are from χ^2^ test, or Mann–Whitney U test

The median time from illness onset to admission was 5 days in the derivation cohort and 7 days in the validation cohort. All patients were isolated after admission, in the derivation cohort, 34 (55.7%) patients received antiviral treatment, of which eight patients received oseltamivir (75 mg every 12 h, orally) and 26 (42.6%) patients received lopinavir and ritonavir tablets (200 mg twice daily, orally). Nearly half of the patients (26, 42.6%) in the derivation cohort received antibiotic therapy. One patient received methylprednisolone for 3 days before admission and stopped using this drug after admission at the hospital, another patient had been taking methylprednisolone 8 mg every other day for 10 months to treat optic neuromyelitis and continued taking it after admission. 20 (32.8%) patients in the derivation cohort received oxygen support and 52 (85.2%) received nebulization inhalation therapy, three patients among these received non-invasive ventilation and two received invasive mechanical ventilation. Nebulization inhalation drugs included recombinant human interferon α2b and acetylcysteine. By the end of Jan 31, no patients had died, three patients were discharged, and the remaining patients were in hospital, of which eight patients progressed to critical illness and received treatment in the ICU (Table 1).

**Fig. 1:**
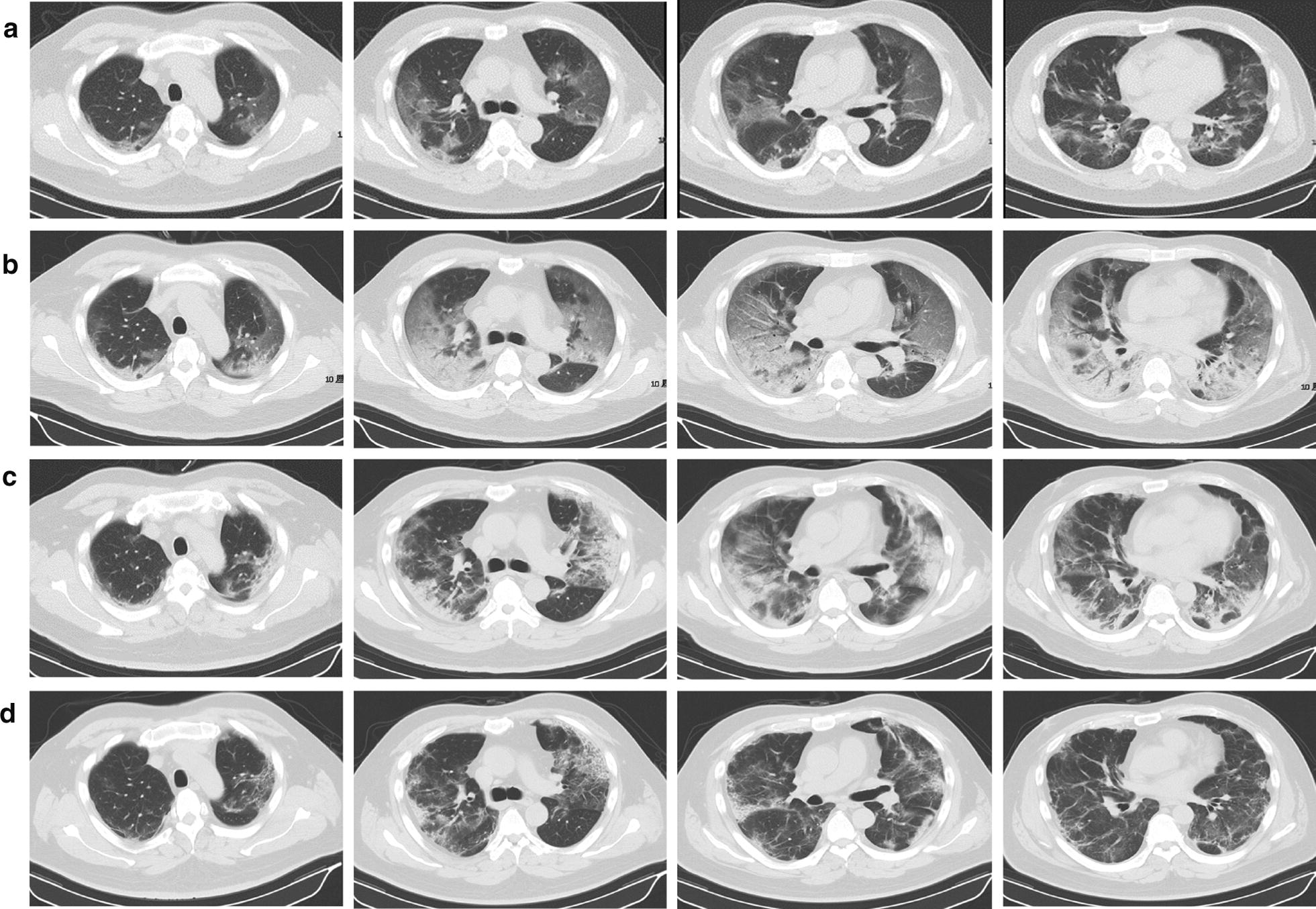
A 50-year-old man with 2019 novel coronavirus (COVID-19) infection. **a** Ground glass shadow in multiple lobes and segments of bilateral lungs; the lesions were adjacent to the pleura (Illness Day 8, Hospital Day 0). **b** Ground glass shadow expanding and consolidation in bilateral lung (Illness Day 11, Hospital Day 3). **c** Ground glass shadow absorption and reduced consolidation area (Illness Day 15, Hospital Day 7). **d** Lesion dissipation (Illness Day 20, Hospital Day 12)

X-ray or CT showed multiple lung lobe or bilateral involvement in 48 (78.7%) patients. Figure 1 showed the CT images of a typical patient in early, consolidation, absorption and dissipation stages.

### Predictive factors of critical illness

**Fig. 2:**
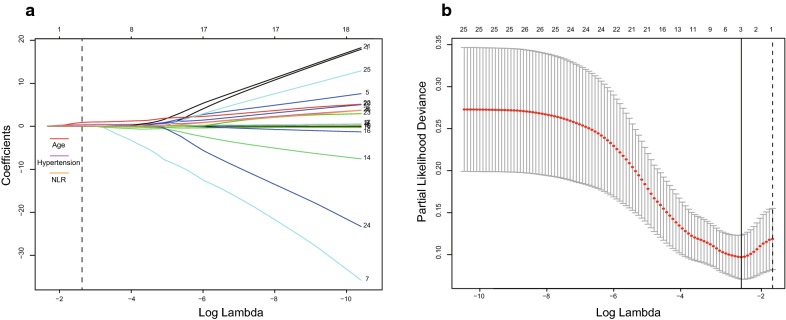
The predictive factor neutrophil-to-lymphocyte ratio (NLR) was selected using LASSO regression analysis. **a** LASSO coefficient profiles of the non-zero variables of COVID-19 pneumonia. **b** Partial likelihood deviance plot of the lowest point of the red curve (solid line), which corresponds to a three-variable model. The dashed line on the right is a more concise model within one standard error (the number of variables is one)

Twenty-six variables were included in the LASSO regression analysis. The variables were demographic status (sex, age, smoking, and drinking history), comorbidity (diabetes, hypertension, and chronic obstructive pulmonary disease [COPD]), CT scan (multiple lung lobe or bilateral involvement), timeline after onset of illness (days from illness onset to admission time), routine blood tests (white blood cell count, neutrophil count, lymphocyte count, monocyte count, NLR, hemoglobin levels, and platelet count), liver function (alanine aminotransferase, albumin, prothrombin time), serum electrolytes (potassium, sodium, and chlorine), kidney function (serum urea nitrogen and creatinine), serum glucose, and C-reactive protein. Age, NLR, and hypertension of the 61 individuals in the cohort were prognostic factors for critical illness incidence when the partial likelihood deviance was the smallest; NLR was the significant predictive factor when the lambda was 1 standard error (Fig. 2a, b). The three factors mentioned above were included in the multivariate COX regression analysis, and the results indicated that age and NLR are prognostic factors for critical illness of COVID-19 infection. However, when the hazard ratios (HR) of age was close to 1, age was transformed into a categorical variable (< 50 years/≥ 50 years) based on cutoff value, and then the three variables were included in the COX regression analysis again. Finally, NLR was selected as the most useful predictive factor for predicting critical illness incidence.

### Nomogram establishment and validation

**Fig. 3:**
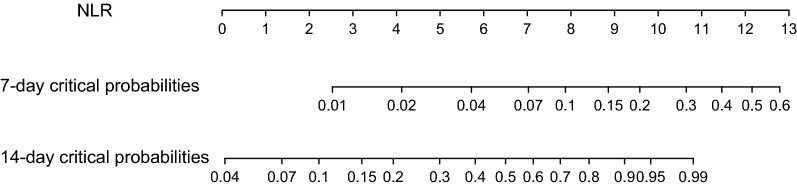
Nomogram predicting 7-day and 14-day critical probability of patients with COVID-19 pneumonia

**Fig. 4:**
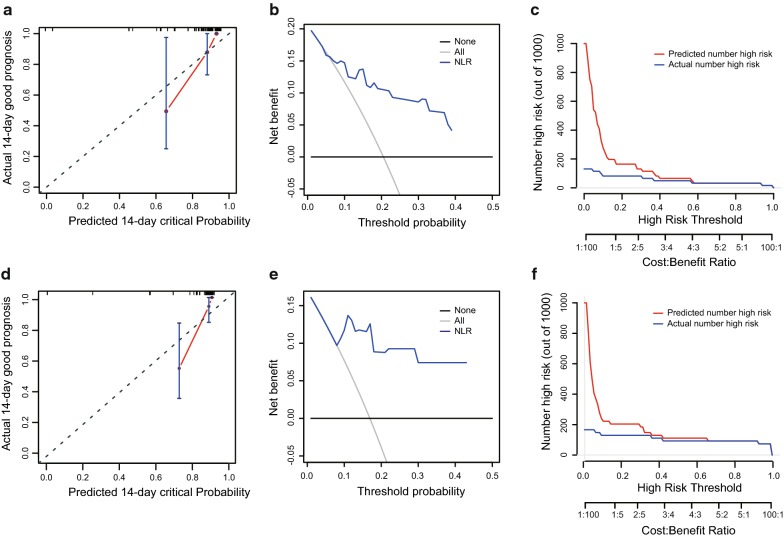
Evaluate the prediction effect of nomogram in the derivation (**a**–**c**) and velidation (**d**–**f**) cohorts. **a**, **d** Calibration plot, **b**, **e** decision curve and **c**, **f** clinical impact curve of the nomogram for critical probability in the COVID-19 cohort, in which the predicted critical probability was compared well with the actual probability and had superior standardized net benefit

The nomogram was established based on the NLR values, which were used to predict the critical rates of 7 and 14 days (Fig. 3). The nomogram had a concordance index (c-index) of 0.807 (95% confidence interval [CI] 0.676–0.938) for predicting the critical probability in the derivation cohort and 0.882 (95% CI 0.778–0.986) in the validation cohort. The calibration curves showed that the predicted rates were in agreement with the actual results observed in the derivation and validation cohorts (Fig. 4a, d). The vertical lines on the upper side reflect the distribution of the predicted probability in patients. The decision curve and clinical impact curve showed that the NLR had superior standardized net benefit and influence on the patient outcome (Fig. 4b, c, e, f).

**Fig. 5:**
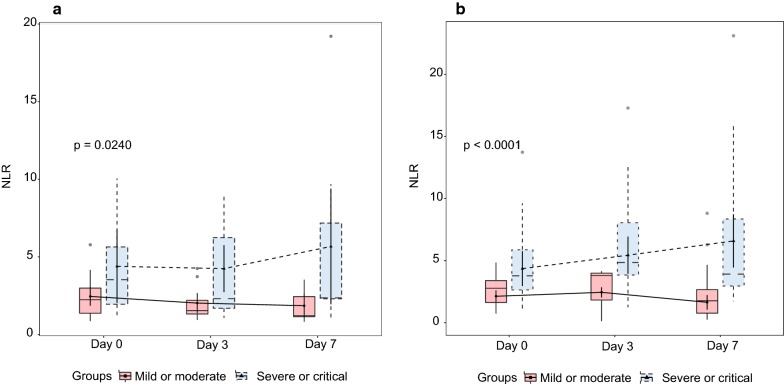
Time-dependent changes in NLR levels in the mild, moderate, and severe or critical groups. The NLR was higher in the severe or critical group, and a significant difference in the decline rate was observed between the two groups (p = 0.0240 and p < 0.0001 for derivation and validation cohorts, respectively)

The NLR values of the patients on the day of admission our hospital and on days 3 and 7 after admission were checked. Figure 5 shows the dynamic changes at different times in patients with COVID-19 classified in the mild or moderate, and severe or critical groups. The NLR values were higher in the severe or critical group on admission and increased more rapidly compared to those in the mild or moderate group (p = 0.0240 and p < 0.0001 for derivation and validation cohorts, respectively).

### Comparison NLR with other models

Using receiver operating characteristic analysis, the predictive value of the NLR for the incidence of critical illness was compared to that of the MuLBSTA [11] and CURB-65 [12–14] models. NLR had the highest area under receiver operating characteristic curve (AUC) (0.849, 95% CI 0.707–0.991), and had higher sensitivity and specificity compared to those of the other two models in the derivation cohort (Table 2). In the validation cohort, the AUC of NLR was 0.867 (95% CI 0.747–0.944), the sensitivity was 0.667 (95% CI 0.299–0.925), and the specificity was 0.978 (95% CI 0.882–0.999).

**Table 2:** Predictive value of the NLR, MuLBSTA and CURB-65

	AUC (95% CI)	c-index (95% CI)	SEN (95% CI)	SPE (95% CI)	PPV (95% CI)	NPV (95% CI)	DLR positive (95% CI)	DLR negative (95% CI)
NLR	0.849 (0.707–0.991)	0.807 (0.676–0.938)	0.875 (0.473–0.997)	0.717 (0.577–0.832)	0.318 (0.200–0.955)	0.974 (0.823–0.987)	3.092 (1.871–5.109)	0.174 (0.028–1.100)
MuLBSTA	0.762 (0.585–0.938)	0.771 (0.659–0.883)	0.875 (0.473–0.997)	0.679 (0.537–0.801)	0.292 (0.184–0.949)	0.973 (0.822–0.986)	2.728 (1.703–4.370)	0.184 (0.029–1.162)
NLR-MuLBSTA	0.851 (0.740–0.963)	0.837 (0.741–0.933)	1.000 (0.631–NA)	0.679 (0.536–0.801)	0.320 (0.205–NA)	1.000 (0.885–1.000)	3.118 (2.107–4.613)	0.000 (0.000–NA)
CURB-65	0.700 (0.505–0.896)	0.744 (0.573–0.915)	0.625 (0.245–0.915)	0.755 (0.617–0.862)	0.278 (0.168–0.712)	0.930 (0.722–0.965)	2.548 (1.247–5.208)	0.497 (0.200–1.232)
NLR-CURB-65	0.889 (0.743–1.036)	0.870 (0.762–0.978)	0.875 (0.473–0.997)	0.868 (0.747–0.945)	0.500 (0.310–0.978)	0.979 (0.855–0.992)	6.63 (3.17–13.86)	0.144 (0.023–0.904)

*AUC* area under curve, *SEN* sensitivity, *SPE* specificity, *PPV* positive predictive value, *NPV* negative predictive value, *DLR* diagnostic likelihood ratios

After NLR was incorporated into MuLBSTA (NLR-MuLBSTA) and CURB-65 (NLR-CURB-65) models by adding the NLR value directly to the score of these two models, respectively, it was found that the prediction effect of the improved model was significantly better than that of the original model, but there was no significant difference between the AUC of NLR and those of NLR-MuLBSTA and NLR-CURB-65 (p = 0.9675 and p = 0.2971, respectively) (Table 2).

### Stratifying patients according to risk

**Fig. 6:**
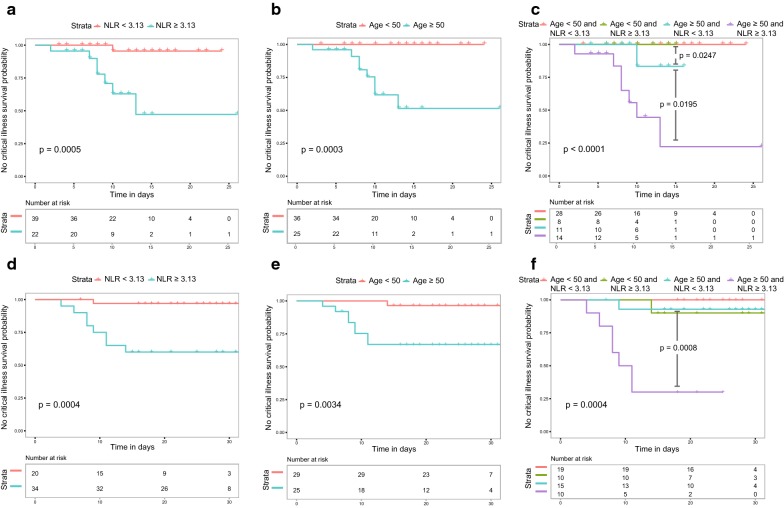
Kaplan–Meier curves of risk group stratification for no critical illness in the derivation cohorts. **a** The cutoffs of NLR for each risk group were as follows: low risk: < 3.13, and high risk: ≥ 3.13. **b** Risk group stratification according to age and **c** NLR combined with age

The median follow-up time was 10 days, minimum 2 days and maximum 26 days. Patients were divided into two strata according to the cutoff value of NLR (low risk: < 3.13; high risk: ≥ 3.13) and age (age < 50 years; age ≥ 50 years). In the derivation cohort, progression to critical illness occurred in 2.6% (1/39) patients in the NLR < 3.13 strata, 31.8% (7/22) in the NLR ≥ 3.13 strata (p = 0.0005, Fig. 6a), 0% (0/36) in the age < 50 years strata, 32% (8/25) in the age ≥ 50 years strata (p = 0.0003, Fig. 6b). In the validation cohort, progression to critical illness occurred in 2.9% (1/34) patients in the NLR < 3.13 strata, 40% (8/20) in the NLR ≥ 3.13 strata (p = 0.0004, Fig. 6d), 3.4% (1/29) in the age < 50 years strata, 32% (8/25) in the age ≥ 50 years strata (p = 0.0034, Fig. 6e). Furthermore, patients with COVID-19 pneumonia were stratified according to age and the NLR (strata 1: age < 50 years and NLR < 3.13; strata 2: age < 50 years and NLR ≥ 3.13; strata 3: age ≥ 50 years and NLR < 3.13; strata 4: age ≥ 50 years and NLR ≥ 3.13). In the derivation cohort, there was no critical illness case in strata 1 (0/28) and strata 2 (0/8); there was 9.1% (1/11) critical illness case in strata 3 and 50% (7/14) critical illness cases in strata 4. As shown in Fig. 6c, the critical illness incidence was significantly different between strata 3 and 4 (p = 0.0195) and between strata 2 and 3 (p = 0.0247). In the validation cohort, there was no critical illness case in strata 1 (0/19); there was 10% (1/10), 6.7% (1/15), and 70% (7/10) critical illness case in strata 2, 3, and 4. As shown in Fig. 6e, the critical illness incidence was significantly different between strata 3 and 4 (p = 0.0008), but the difference between strata 2 and 3 was not significant (p = 0.8317).

## Discussion

Since the outbreak of the COVID-19 pneumonia in December 2019, there have been 2000 to 4000 new confirmed cases of infection every day in China, and the number of severe cases and deaths has also been increasing day by day. Recent research showed that 26% of patients received ICU care, and mortality was 4.3% [15]. The number of patients in Wuhan and other regions is increasing rapidly. The current difficulty is the shortage of medical resources, especially critical care resources. Early identification critical illness and risk stratification management will help alleviate insufficient medical resources and might reduce mortality. Recent studies have reported that low lymphocyte-to-C-reactive protein ratio [16], platelet-to-lymphocyte ratio [17], and thrombocytopenia [18] may be associated with critical illness. In addition, smoking and COPD have been associated with COVID-19 [19]. These may not have influenced the results in the present study because of the low number of subjects (a total of 10 smoking and 6 COPD patients).

The COVID-19 pneumonia is not severe in the early stage, but the critical patients deteriorated on 7–14 days of illness course and entered a state of severe pneumonia and acute respiratory failure. The critical or death patients with COVID-19 infection were mostly of an old age and had comorbidities [20]. In the study, the critical ill patients were all over 50 years old. The decrease of lymphocyte count was related to the progress of the disease. It is unclear why lymphopenia is associated with severe illness. It has been hypothesized that COVID-19 may act on T lymphocytes, and T lymphocyte damage is an important factor that causes deterioration of the patient’s condition [21]. In addition, a high leukocyte count is common in critically ill patients because damaged cells induce innate inflammation in the lungs, which is largely mediated by proinflammatory macrophages and granulocytes [22]. The NLR was a widely used marker for the assessment of the severity of bacterial infections and the prognosis of patients with pneumonia and tumors [21, 23–28].

In this study, the data of 115 patients with COVID-19 pneumonia were analyzed, the baseline characteristics of patients in the derivation and validation cohorts were described and compared, and the dynamic changes of laboratory indexes and imaging features were demonstrated. The independent risk factors affecting incidence of critical illness were screened. The results showed that NLR was the most important prognostic factor for progression, followed by age. Furthermore, according to the NLR and age stratification, the incidence of critical ill patients with NLR ≥ 3.13 and aged ≥ 50 years was 50%, and 9.1% in aged ≥ 50 years and NLR < 3.13 patients.

Previous studies showed that the MuLBSTA score can give an early warning regarding the mortality of viral pneumonia; this score includes six indicators, namely, age, smoking history, hypertension, bacterial co-infection, lymphopenia, and multilobular infiltration [11]. The CURB-65 score was widely used to evaluate 30-day mortality of patients with community-acquired pneumonia [12–14]. In the study, NLR was compared with MuLBSTA and CURB-65 scoring models. The results showed that NLR had higher AUC, c-index, sensitivity and specificity, which indicated that NLR was better than the other two models for predicting the early incidence of COVID-19 critical illness. Furthermore, it was found that the prediction effects of the NLR-MuLBSTA and NLR-CURB-65 models were better than those of the original models. But NLR was an easy-to-use predictor index.

**Fig. 7:**
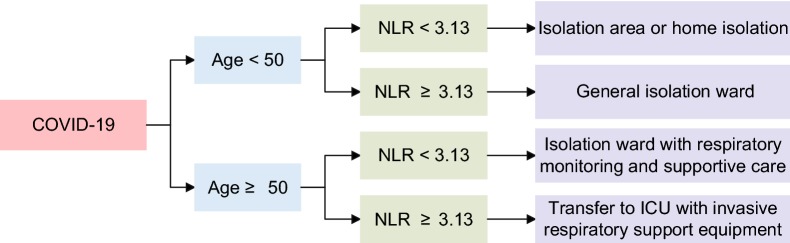
COVID-19 pneumonia management process

The risk stratification of NLR according to age facilitates patient management. Patients aged < 50 years with an NLR < 3.13 highly unlikely to develop a critical illness and can be treated in a community hospital or home isolation; patients with NLR ≥ 3.13 have a low chance of developing a critical illness need to be treated in a general isolation ward and closely monitored. Patients aged ≥ 50 and having an NLR < 3.13 have a moderate chance of developing a critical illness, and admitting to isolation ward with respiratory monitoring and supportive care was needed for these patients; patients aged ≥ 50 and having an NLR ≥ 3.13 have a high risk of developing a critical illness and need to be prepared for transfer to ICU for invasive respiratory support equipment (Fig. 7). If there are large-scale cases, the risk stratification and management will help alleviate the shortage of medical resources and reduce the mortality of critical patients.

There were some limitations to the study. First, we performed this study in low number of subjects (61 in the derivation cohort and 54 in the validation cohort). However, after we published our present study at medRxiv (https://medrxiv.org/cgi/content/short/2020.02.10.20021584v1) on February 12, 2020, another group draw similar conclusion by a meta-analysis with 828 patients [16]. The status of patients in the derivation and validation cohorts may be different, leading to an imbalance in some laboratory indicators, such as white blood cell count, neutrophil count, and lymphocyte count. CRP [29], cardiovascular disease [30] and COPD [19] were not associated with COVID-19 in this study. These may be due to the low number of subjects. Future multicenter studies with large sample sizes are needed to explore the applicability of the risk stratification of NLR according to age in predicting the critical illness of COVID-19. Second, most of patients are still in hospital, whose condition maybe change in follow-up, and the study has not included the final survival outcome of patients. However, we focused on the early identification of critical cases for risk stratification and management. We expect that the risk model can help alleviate the shortage of medical resources and manage the patients with COVID-19 pneumonia.

## Conclusion

The NLR was the most promising predictive factor for critical illness incidence of COVID-19 pneumonia. The early application of NLR and age will be beneficial to patient classification management and relief of medical resource shortage.

## Data Availability

The datasets used and/or analyzed during the current study are available from the corresponding author on reasonable request.

## Abbreviations

AUC: Area under receiver operating characteristic curve
COVID-19: 2019 coronavirus disease
CI: Confidence interval
ICU: Intensive care unit
NLR: Neutrophil-to-lymphocyte ratio
